# Switching Attention Within Working Memory is Reflected in the P3a Component of the Human Event-Related Brain Potential

**DOI:** 10.3389/fnhum.2015.00701

**Published:** 2016-01-06

**Authors:** Stefan Berti

**Affiliations:** Department of Clinical Psychology and Neuropsychology, Institute for Psychology, Johannes Gutenberg-University MainzMainz, Germany

**Keywords:** working memory, object switching, memory updating, event-related brain potentials (ERP), P3a, selective attention, controlled attention

## Abstract

The flexible access to information in working memory is crucial for adaptive behavior. It is assumed that this is realized by switching the focus of attention within working memory. Switching of attention is mirrored in the P3a component of the human event-related brain potential (ERP) and it has been argued that the processes reflected by the P3a are also relevant for selecting information within working memory. The aim of the present study was to further evaluate whether the P3a mirrors genuine switching of attention within working memory by applying an object switching task: Participants updated a memory list of four digits either by replacing one item with another digit or by processing the stored digit. ERPs were computed separately for two types of trials: (1) trials in which an object was repeated and (2) trials in which a switch to a new object was required in order to perform the task. Object-switch trials showed increased response times compared with repetition trials in both task conditions. In addition, switching costs were increased in the processing compared with the replacement condition. Pronounced P3a’s were obtained in switching trials but there were no difference between the two updating tasks (replacement or processing). These results were qualified by the finding that the magnitude of the visual location shift also affects the ERPs in the P3a time window. Taken together, the present pattern of results suggest that the P3a reflects an initial process of selecting information in working memory but not the memory updating itself.

## Introduction

The human working memory system comprises of cognitive processes enabling the maintenance and access of relevant information in the service for mental tasks or action control (for instance, see Cowan, 1998). One central function of the working memory system is the selection of relevant information either provided by the sensory input or represented within the cognitive system (i.e., in long term memory; Cowan, 1995). Importantly, the high flexibility of the cognitive system relies on a fast and efficient access to information in working memory, i.e., by switching from one to another memory item. The controlled attention view of working memory (see Cowan, 1998; Engle et al., 1999; Oberauer, 2002; Oberauer et al., 2013) assumes that relevant information is accessed by switching the focus of attention within the memory system. Switching of the focus of attention is also a pre-requisite for selection of relevant information provided by the sensory environment (for instance, see Näätänen, 1992; Johnston et al., 1995; Wright and Ward, 2008). Therefore, the question arises whether these two functions—selection of relevant information in the sensory environment and selection of relevant information in working memory–share some neuro-cognitive processes (for instance, see Awh and Jonides, 2001; Bledowski et al., 2010). This question can be investigated by application of event-related brain potentials (ERPs) by testing whether components typically correlated with processes of attentional orientation in the sensory environment are also associated with performance in working memory tasks (for instance, see Griffin and Nobre, 2003; Berti, 2008; Kuo et al., 2009). In the present study, the association between the P3a component (as an index of attentional allocation to sensory information) and switching within working memory is tested.

**Figure 1:**
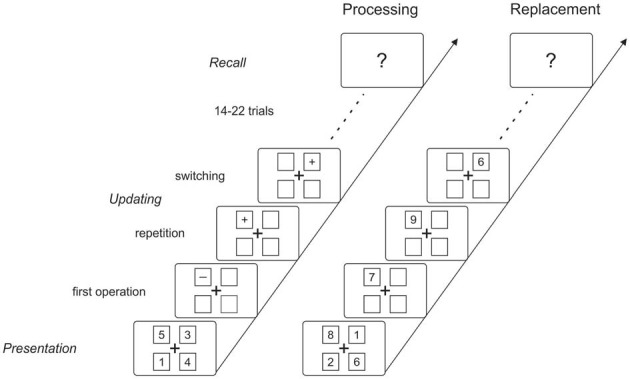
**Stimulation and timing of the stimulus presentation in the two updating tasks.** Both tasks started with the presentation of the memory items (digits from 1–9) in four squares. After the memorization phase, the digits disappeared but the squares remained on the screen during each updating sequence and served as cues for the respective memory item. In the replacement condition, randomly assigned digits were presented in one of the four squares and the participants’ task was to replace the indicated item with the new digit. In the processing condition, either a plus or a minus sign was presented within a square and the participants’ task was to subtract or add one to the indicated memory item. In both conditions, the participants were instructed to update the memory item as fast as possible and to press a button after they completed the updating task. From trial to trial, either the same or another memory item was updated compared with the preceding trials, resulting in two types of trials within each sequence (i.e., switching vs. repetition trials). After each sequence, the participants had to recall the final list of memory items. The number of updating trials within each sequence varied between 17 and 25.

The controlled attention view of working memory assumes that the control of the focus of attention plays a crucial role in selecting relevant information either in the sensory or in the memory system (see Cowan, 1998). This assumption was tested in an ERP study by Berti (2008), applying a working memory updating task (see Oberauer, 2002). The question was whether ERP correlates that mirror allocation of the focus of attention in the sensory domain also mirror the switch of attention between different objects stored in working memory. In more detail, Berti (2008) tested whether the so called P3a—an ERP component assumed to reflect automatic allocation of attention to rare or unexpected information appearing in the physical environment (see Escera et al., 2001; Polich, 2007; in contrast, for the novelty P3, see Friedman et al., 2001; for the distinction between novelty P3 and P3a see Simons et al., 2001)—does also mirror the controlled allocation of attention in a memory updating task. Figure 1 depicts the logic of the updating task (Oberauer, 2002; for other variants, see Morris and Jones, 1990; Garavan, 1998; Kessler and Meiran, 2008) which consists of three phases: In the presentation phase, numbers are presented at different locations on the screen which are memorized by the participant. In the updating phase mathematical calculations (e.g., plus two, minus one) are presented and the participant is instructed to update the respective memory item. More important, on a trial-by-trial basis the relevant object is either repeated or changed: when the calculation is presented at a new location the task requires selecting another item in working memory (i.e., object-switch). In contrast, when the location is repeated no object-switch is required to perform the task. The object-switch is accompanied by increased response times which are assumed to mirror the extra time required for switching the focus of attention to another object in working memory (see Garavan, 1998; Oberauer, 2002; Berti, 2008; see Oberauer et al., 2013, for detailed discussion of this effect). The final phase is the recall of the actual memory list after a number of updating trials.

The present study aims in further evaluating the role of the processes mirrored by the P3a in the context of working memory updating. The results by Berti (2008) suggest that the P3a mirrors control of the focus of attention which is required in the continuous memory updating task when a new object is selected. On the contrary, the Berti (2008) study does not totally rule out that the P3a is correlated with another aspect of memory updating. For instance, according to Ecker et al. (2010) working memory updating consists of three sub-processes, namely retrieval, transformation, and substitution (see also Bledowski et al., 2009). Therefore, it is possible that the P3a mirrors the transformation or the substitution of a memory item instead of the control of attention. To test whether the P3a reflects the switch of the focus of attention as an initial process of working memory updating, two memory updating tasks are applied: the first task replicates the task in Berti (2008; see also Oberauer, 2002) and includes a processing of the stored memory item. The second task only requires replacing the old memory item by a newly presented number but no transformation (for instance, see Morris and Jones, 1990; Oberauer, 2003; Kessler and Meiran, 2008). In both tasks, half of the trials are object-switching trials and the other half of the trials are object-repetition trials.

The following outcome of the experiment is expected: As demonstrated in previous studies (i.e., Oberauer, 2002), object switching is accompanied by an increase in processing time. Therefore, in both conditions of the present experiment object-switching costs are expected. In addition, Berti (2008) demonstrated that object switching is also mirrored by an increased P3a reflecting attentional allocation to the now relevant memory object. This is in line with the assumptions of the controlled attention view of working memory (Cowan, 1998; Engle et al., 1999; Oberauer, 2002; Oberauer et al., 2013). Therefore, in both types of task a pronounced P3a should accompany switching trials. Furthermore, the P3a should not differ between the two types of tasks because it is assumed that the P3a mirrors only the initial stage of allocation of attention (as a pre-requisite for the subsequent task) which does not differ between the two tasks.

## Materials and Methods

### Participants

Twenty students of the Johannes Gutenberg-University Mainz (age range: 19–45 years, mean age: 22.2 years; six male; two left handed) with normal or corrected to normal visual acuity participated in the study. All participants received course credit after completion of the experiment. In accordance with the Declaration of Helsinki, the participants gave written consent after the nature of the study and the procedure were explained to them. All participants reported a normal general and neurological health status. A subset of these participants was selected in order to reveal a high signal-to-noise ratio in the EEG data; this selection was based on two criteria, one based on performance accuracy and one based on EEG data quality (for details, see below). The final group of participants included in data analysis consisted of 13 volunteers (age range: 19–26 years, mean age: 21.2 years; five male; two left handed).

### Task and Procedure

The participants performed a memory updating task in two different conditions. Figure 1 depicts the general procedure of a memory updating sequence: Each updating sequence started with the initial presentation of four digits to be memorized followed by a variable number of updating trials. Each sequence finished with the recall of the resulting memory list after the updating trials. Thus, an updating sequence consists of three phases: the *presentation phase*, the *updating phase*, and the *recall phase*. All stimuli presented in the three phases (including the fixation cross and the squares) were presented in black (RGB code 3.6, 3.5, 2.4; luminance 3.5 cd/m^2^) against a medium gray background (74.2, 79.4, 56.2; 78.2 cd/m^2^).

In the *presentation phase* four squares were presented surrounding a central fixation cross, and within these four squares, digits between 1 and 9 were presented simultaneously. In this phase, participants were instructed to memorize these digits at the specific location for the reason that the location within the frame served as the cue for the subsequent updating trials. The memory list was deleted after 10 s or when the participants finished the memorization by a button press. The frame of the four squares was continuously presented during the updating phase. Moreover, the digits were randomly selected offline with replacement in order to prevent that one digit was presented within a memory list more than once.

In the *updating phase*, either a digit or a plus or minus sign (depending on the respective condition) was presented within one of the four squares. In this phase, the participants’ task was to update the memory item indicated by the respective square and to press a key when they finished the updating procedure. The time for the memory updating was restricted to 10 s. After this time or after the participant pressed a response button the next operation was displayed with a 500 ms asynchrony. In this phase, participants were instructed to update the memory list as fast and as accurately as possible and especially to press the response button as quickly as possible after updating the memory list for the reason that–in addition to accuracy of memory recall–response times during memory updating served as a measure of task performance. The updating phase differed with regard to the number of trials, ranging from 17–25 with an overall average of 20 updating trials.

After the final updating trial, the *recall phase* started: In this phase, the participants’ task was to recall aloud the final memory list as accurately as possible; an experimenter wrote down the participants’ answers.

The two conditions differed only with regard to the type of updating procedure within the updating phase; the general procedure of the memory updating sequence was the same in both conditions. In the *replacement condition* (see Figure 1, right), a digits between 1 and 9 was presented within one position of the frame (i.e., within one of the four squares) and the participants were instructed to replace the respective memory item by the currently presented digit. In the *processing condition* (Figure 1, left), either a plus or a minus sign was presented within one position of the frame and the participants were instructed to calculate the new memory item (i.e., either adding one or subtracting one to/from the respective memory item) and to memorize the result instead of the original digit. Each memory updating sequence required either replacement or processing of the memory item. In both conditions, the presentation of each digit or sign were equally likely and the respective sign/digit was not predictable. In addition, the participants were instructed that the intermediate as well as the final memory lists contained only numbers in the range of 1–9.

In both conditions the updating procedure could either apply to a position that was already updated in the preceding trial or to a position that was not updated in the preceding trial. In other words, in case that the location for the updating was switched compared with the preceding trial, a new memory item had to be activated or retrieved in order to perform the updating task; these trials are referred to as *switching trials* because the relevant memory item changed. In contrast, in trials in which the currently relevant memory item was repeated, no switch was required to perform the task; these trials are referred to as *repetition trials*. Switching and repetition trials were equally likely within the experiment. For the reason that repetition and switching trials are assigned with reference to the preceding trial, the first trial of each sequence cannot be classified in this way and therefore, was not analyzed further.

### General Procedure

The study consisted of two parts, a training session and an experimental session: In the *training session*, participants were instructed and practiced the two updating tasks. In order to increase task performance, participants received direct feedback on whether the recall after each sequence was correct or not. In addition to task performance, eye-movements were monitored via the electro-occulogram (EOG) and after completion of each sequence participants also received a feedback about their eye-movements (i.e., whether they managed to reduce the number of eye-movements or blinks) in order to practice fixating their eyes on the center of the screen (i.e., to the fixation cross). The training session consisted of six memory updating sequences per condition. At the beginning of the training session, the participants were informed about the nature of the general study and instructed about the two different tasks. In detail, the instructions stressed two aspects of task performance: First, accuracy in memory recall at the end of each sequence and second, speed of performing the memory updating during each sequence. In other words, participants were aware that two measures of their performance were relevant for the experiment. Total duration of the training session was 1 h. In the experimental session, participants were prepared for the EEG recording and received a short recapitulation of the task instructions. The *experimental session* consisted of 12 memory updating sequences for each condition resulting in 240 updating trials per condition; participants received no feedback about their task performance after completion of a sequence. The duration of the experimental session was 3 h per participant. The participants performed the training session and the experimental session on two different days; on average, participants completed the experimental session 4 days after the training session (ranging between 1 and 9 days).

### Initial Data Analysis

Data analysis started with the evaluation of the subjects’ performance within the memory task. The main aim of the study was to unravel the ERP correlates of the switch of the focus of attention in working memory. For this reason, only memory updating sequences with error free performance in the recall of the memory list were included in further analysis of RT data and in computation of the ERPs; participants with less than five sequences with correct memory recall in one or both conditions were excluded from data analysis in general. In addition, two further participants were excluded from data analysis for the reason of a too low number of artifact free EEG epochs (i.e., fewer than 40 epochs in one or more trial types) due to a high rate of eye movements. As noted above, on the basis of this procedure 13 participants were included into the final dataset.

### Analysis of Behavioral Data

Based on the data of these 13 participants, the *updating time* (UT) was computed. The UT was defined as the time a participant needed to update the respective memory item (i.e., either by processing or by replacement); UT was measured from the presentation of the digit or the plus/minus sign on the screen to the time when the participant pressed the button to indicate that he or she performed the required operation. Mean UTs for switching and repetition trials were computed separately for the replacement and the processing condition in error free sequences. Moreover, the first trial of a memory updating sequence and trials with an UT shorter than 300 ms were excluded from mean UT computation. To test for effects of the type of trial and the condition, UT was analyzed by means of a repeated-measurement analysis of variance (ANOVA) with the factors *Condition* (replacement vs. processing) and *Trial type* (switching vs. repetition); partial Eta spared (${\text{η}}_{p}^{2}$) is reported as a measure of effect-size for the ANOVA results.

### Electroencephalographic Recording and Analysis

During the experiment, the electroencephalogram (EEG) was recorded using a Synamps amplifier (Neuroscan, Virginia, USA) from nine cap-mounted electrodes (Easy-Cap, FMS, Munich, Germany) of the 10–20 system (F3, Fz, F4, Cz, P3, Pz, P4, O1, and O2); the reference electrode was placed on the right mastoid. The EEG was recorded with a sampling rate of 500 Hz with an online 0.05–70 Hz band pass and a 50 Hz notch filter. In addition, the vertical and horizontal EOG was recorded to control for eye-movements. The EEG was filtered offline with a 30 Hz low-pass. ERPs were computed for switching and repetition trials separately for both conditions within a −200–600 ms time window relative to the onset of the presented operation. The 200 ms pre-stimulus interval served as a baseline. Epochs with extensive EOG activity (i.e., whenever the standard deviation within a 200 ms interval exceeded 25 μV in the EOG) as well as the first operation within a memory updating sequence were excluded from ERP computation. This procedure resulted in a minimum of 43 epochs and a maximum of 118 epochs as basis for individual ERP computation. The mean number of epochs for computation of the ERPs were in the replacement condition 84.8 (repetition) and 85.2 (switching) and in the processing condition 69.9 (repetition) and 69.8 (switching). The difference between the two conditions, however, did not reach significance on the 5% level in a 2 × 2 repeated-measurement ANOVA: *Condition*: *F*_(1,12)_ = 3.45, *p* = 0.088, ${\text{η}}_{p}^{2}$ = 0.22; *Trial type*: *F*_(1,12)_ < 1; *Condition* × *Trial type*: *F*_(1,12)_ < 1.

According to the hypotheses, the analysis of the ERP data focused on the P300 time window at midline electrodes (Fz, Cz, and Pz). After visual inspection of the grand average ERPs, mean amplitudes were calculated at the three midline electrodes within two consecutive 100 ms time windows (230–330 ms and 330–430 ms) in order to tap the complete P300 component. To test for different effects of the trial type in the two task conditions, a four-way repeated-measurement ANOVA with the factors *Condition* (replacement vs. processing), *Trial type* (switching vs, repetition), *Electrodes* (Fz, Cz, and Pz), and *Time window* (early vs. late window) was computed. Further statistical analyses were applied for the two time windows separately to unravel the significant three-way interaction. For the reason that the factor condition revealed no significant effects in the initial ANOVA, the ERP data were pooled for the two types of tasks. A 2 (*Trial type*) × 3 (*Electrode*) ANOVA was calculated for the early and the late time window separately. For all ANOVA results partial Eta spared (${\text{η}}_{p}^{2}$) is reported as a measure of effect-size. In addition, for all effects with two or more degrees of freedom in the numerator, the Greenhouse-Geisser correction was applied.

### *Post hoc* Analysis of the ERP Data

One characteristic of the applied memory updating task is that the factor trial type is confounded with a spatial factor. In trials in which the particular memory item for the updating procedure has to be switched, the cue for the subsequent updating procedure is presented at a location differing with regard to the distance from the current to the new spatial location. In order to test whether this spatial factor may affect the ERPs obtained in the switching trials, a *post hoc* analysis was computed. In this analysis, switching trials in both conditions were separated into near- and far-switches: near-switches were defined as switches between horizontal neighbors and far-switches were defined as switches between diagonally opposite positions (note that both types of trials require a switch to the other visual hemifield). This distinction mirrors that horizontally adjacent stimuli were separated by 4.1° and diagonally opposite stimuli were separated by 6.9°. To increase the signal-to-noise ratio, near- and far-switch trials of both task conditions were pooled together. Again, only ERP epochs from error free memory updating sequences were analyzed. The statistical analysis of the effects was restricted to the early phase at the Cz electrode because the results in the later time window mirrors the pattern of results of the initial analysis.

## Results

**Figure 2:**
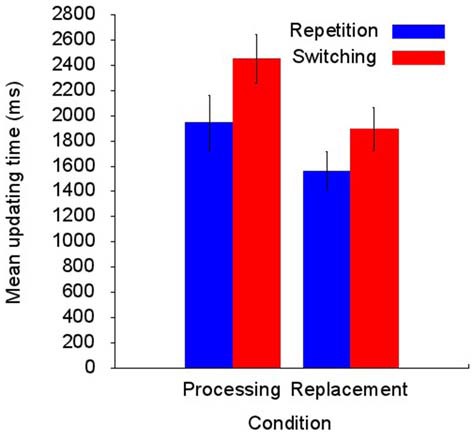
**Summary of the processing times required to perform the updating task (*N* = 13).** The updating time (UT) is longer in the processing than in the replacement condition and is increased in switching compared with repetition trials. In addition, the increased UT for switching trials is more pronounced in the processing condition compared with the replacement condition (for details see text).

The mean of correctly recalled memory lists was 9.5 (95% CI [8.6, 10.3]) in the replacement condition and 7.8 (95% CI [6.5, 9.0]) in the processing condition. This suggests higher task demands in the processing compared with the replacement condition. However, this difference did not reach statistical significance on a 5% level: *t*_(12)_ = 1.97, *p* = 0.072, Cohen’s *d* = 0.55. Figure 2 summarizes the UT results showing longer UTs in the processing condition compared with the replacement condition and longer UTs in switching compared with repetition trials. This pattern of results is mirrored by the ANOVA in significant main effects of *Condition*, *F*_(1,12)_ = 27.28, *p* < 0.001, ${\text{η}}_{p}^{2}$ = 0.69, and *Trial type*, *F*_(1,12)_ = 29.79, *p* < 0.001, ${\text{η}}_{p}^{2}$ = 0.71. This finding is qualified by a significant interaction term, *F*_(1,12)_ = 5.23, *p* = 0.041, ${\text{η}}_{p}^{2}$ = 0.30, showing that the prolonged UT for switching trials is increased in the processing condition compared with the replacement condition. Mean switching costs (i.e., switching UT minus repetition UT) in the replacement condition was 333 ms (95% CI [153, 513]) and 505 ms (95% CI [313, 698]) in the processing condition, *t*_(12)_ = 2.29, *p* < 0.041, *d* = 0.63, 95% CI [8, 336].

**Figure 3:**
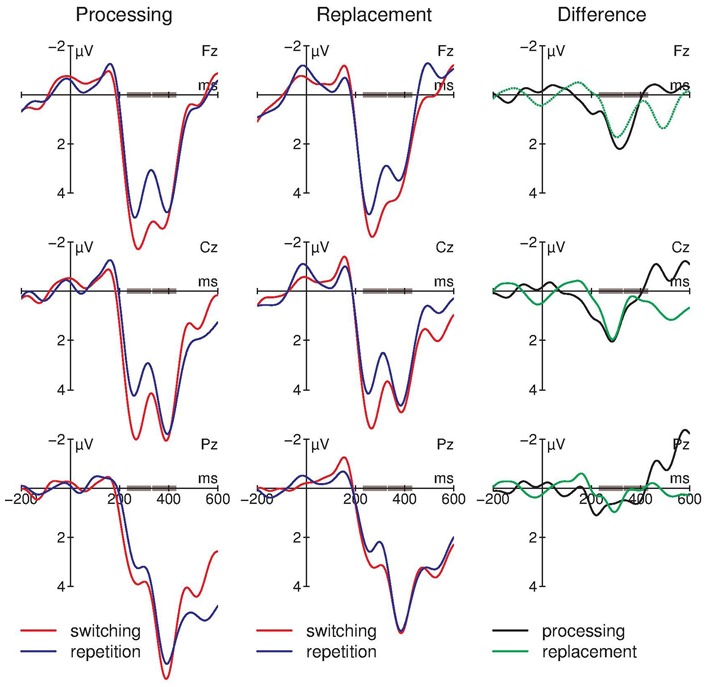
**Summary of the event-related brain potential (ERP) results at midline electrodes (*N* = 13).** In the processing (left column) and in the replacement (middle column) condition a bi-phasic positive component between 200 and 400 ms is observable in both trial types. The early phase of the component peaks at the fronto-central electrodes; this phase resembles the P3a. The later phase of the component peaks at the parietal electrode, resembling the P3b. In addition, the difference waves (ERPs in switching minus ERPs in repetition trials; right column) demonstrate that in both task conditions switching trials elicited a bigger P3a compared with repetition trials (see the positive difference at Fz). In contrast, no differences between the tasks or the trial types are observable in the P3b time window (see Fz). For graphical display, the ERPs were filtered with a 10 Hz low-pass filter; the small gray rectangles embedded in the time-axis mark the early and late P300 time windows.

Figure 3 summarizes the ERP results as obtained at the three midline electrodes. In both conditions, a bi-phasic positive component between 200 ms and 500 ms is visible with a first peak around 260 ms that is maximal at Fz and a second peak around 380 ms that is maximal at Pz. Within the early time window, in switching trials the positive ERPs are enhanced compared with repetition trials. In the right column of Figure 3 the difference between switching and repetition trials in both task conditions is summarized by means of different waves (i.e., ERPs in switching trials minus ERPs in repetition trials). In both conditions, switching trials elicited more positive ERPs around 300 ms at fronto-central leads.

Table 1 summarizes the statistical results obtained with the four-way repeated-measurement ANOVA: this analysis reveals highly significant interactions of Electrode and Time window as well as Trial type, Electrode, and Time window; no effect of Condition was obtained. The subsequent 2 × 2 *post hoc* ANOVAs revealed an effect of Trial type in the early time window only: 230–330 ms window: *Trial type*: *F*_(1,12)_ = 4.51, *p* = 0.055, ${\text{η}}_{p}^{2}$ = 0.27; *Electrode*: *F*_(2,24)_ = 6.03, *p* = 0.020, ${\text{η}}_{p}^{2}$ = 0.34; *Trial type* × *Electrode*: *F*_(2,24)_ = 4.43, *p* = 0.027, ${\text{η}}_{p}^{2}$ = 0.27; 330–430 ms window: *Trial type*: *F*_(1,12)_ = 1.58, *p* = 0.233, ${\text{η}}_{p}^{2}$ = 0.116; *Electrode*: *F*_(2,24)_ = 7.04, *p* = 0.011, ${\text{η}}_{p}^{2}$ = 0.37; *Trial type* × *Electrode*: *F*_(2,24)_ < 1. Table 2 summarizes the amplitude difference between switching and repetition trials (compare Figure 3, right column) separately for the early and late time window and the three electrodes. This suggests that the Trial type-by-Electrode interaction in the early time window is due to a substantial positive difference between switching and repetition trials at fronto-central electrodes but not at the parietal lead; no general positive effect for switching trials is obtained in the late time window. As depicted in Figure 3 (left and middle columns), the main effect of electrode in the late time window reflects the increase of the positivity from frontal to parietal leads.

**Table 1:** **Statistical evaluation of the effects of Condition (processing vs. replacement), Trial type (switching vs. repetition), Electrodes (Fz, Pz, and Cz), and Time window (230–330 ms vs. 330–430 ms) on the ERP results by means of a repeated-measurement ANOVA (*N* = 13)**.

Factor	*df*	*F*	*p*	${\text{η}}_{p}^{2}$
Condition (C)	1, 12	3.37	0.091	0.22
Trial type (T)	1, 12	3.62	0.081	0.23
Electrode (E)	2, 24	<1	0.937	<0.01
Time window (W)	1, 12	1.95	0.188	0.14
C × T	1, 12	<1	0.728	0.01
C × E	2, 24	1.54	0.239	0.12
T × E	2, 24	1.94	0.177	0.14
C × W	1, 12	1.61	0.229	0.12
T × W	1, 12	2.89	0.114	0.19
E × W	2, 24	54.89	<0.001	0.82
C × T × E	2, 24	<1	0.824	<0.01
C × T × W	1, 12	<1	0.983	<0.01
C × E × W	2, 24	<1	0.685	0.03
T × E × W	2, 24	9.77	<0.001	0.45
C × T × E × W	2, 24	1.35	0.269	0.11

*Note: Greenhouse-Geisser correction was applied; uncorrected degrees of freedom and corrected p-values are reported. df = degrees of freedom*.

**Table 2:** **Mean amplitude of the difference waves (ERPs in switching trials minus ERPs in repetition trials) in the early (230–330 ms) and late (330–430) time window (*N* = 13)**.

Time window	230–330 ms		330–430 ms	
Electrode	Mean amplitude (μV)	95% CI	Mean amplitude (μV)	95% CI
Fz	1.26	[0.06, 2.46]	0.73	[−0.22, 1.68]
Cz	1.54	[0.19, 2.89]	0.36	[−0.42, 1.14]
Pz	0.67	[−0.51, 1.85]	0.30	[−0.68, 1.27]

*Note: the mean amplitudes were derived from ERPs pooled for the two task conditions. CI = confidence interval*.

**Figure 4:**
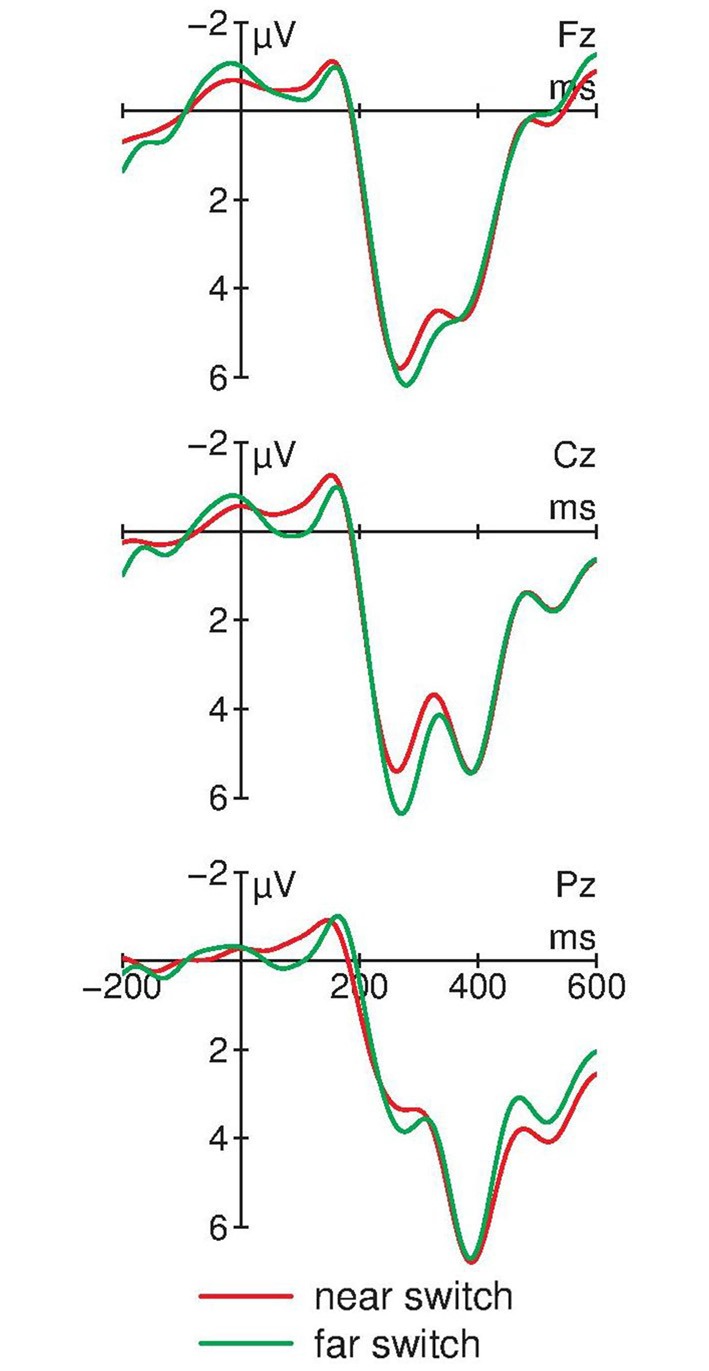
**ERPs for far-switches and near-switches at midline electrodes (*N* = 13).** Near- and far-switches of attention again show the bi-phasic pattern of positive components between 200 and 400 ms. Differences between these different types of switching trials are observable between 250 and 330 ms at Cz with an attenuated positive amplitude for far-switches compared with near-switches. For graphical display, the ERPs were filtered with a 10 Hz low-pass filter.

Figure 4 compares the ERPs in trials with a switch to horizontally adjacent positions (near-switch) compared with switches to diagonally opposite positions (far-switch). In both switching trial types, two positive components between 200 and 400 ms are elicited. In addition, far-switches obtain a more pronounced positivity around 250 ms at fronto-central electrodes. A one-way repeated measure ANOVA of the mean amplitudes between 250 and 330 ms at Cz obtains a significant effect of the factor *Switching type*: *F*_(1,12)_ = 4.96, *p* = 0.046, ${\text{η}}_{p}^{2}$ = 0.29.

## Discussion

Three main outcomes emerged from the analysis of behavioral and electrophysiological data. First, switching trials are accompanied by an increased positive ERP component peaking around 300 ms at fronto-central electrodes (best visible in the difference waves). Secondly, an object switch increased the time the participants required for memory updating. Third, while the updating costs varied between the two updating tasks, the increased frontal positivity did not vary between task conditions. In addition, updating of memory items is also accompanied by a positive component peaking around 400 ms at Pz; this positive ERP component does not vary with the type of trial or task. With regard to the timing and the fronto-central distribution, the first positive peak depicts the P3a (Escera et al., 2001; Polich, 2007); the later positivity with parietal distribution can be identified as P3b (Donchin and Coles, 1988; Polich, 2007). Finally, the *post hoc* analysis of the switching trials also obtained an effect of the distance between the spatial locations on the switching ERPs.

Selecting a new item in working memory for subsequent processing or replacement requires time. One factor–besides other potential factors–is that the focus of attention has to be allocated to the relevant item (cf. Oberauer, 2002). The finding that the P3a is increased in switching trials compared with repetition trials supports this interpretation because the P3a reflects allocation of attention (e.g., Berti and Schröger, 2003; Polich, 2007). The present findings are also in line with recent behavioral (e.g., Oberauer, 2002; Kessler and Meiran, 2008) and electrophysiological (Berti, 2008) studies. In addition to the Berti (2008) study, the present results demonstrate that the type of memory updating procedure (i.e., processing vs. replacement) does not affect the P3a effects. This supports the interpretation that primarily the allocation of attention to an item in working memory is mirrored in the P3a. A subsequent phase of working memory updating is mirrored in the later P3b component, which is unaffected by different aspects of the task instructions (i.e., switching vs. repetition and replacement vs. processing). This can be interpreted in line with the assumption that the P3b reflects processes involved in working memory updating (Donchin and Coles, 1988). In the present study, the amount of information that is relevant and updated is always the same (i.e., one item out of a list of four items). Therefore, the finding that the P3b does not differ between trial types and condition may mirror the constant amount of information processed or transferred in working memory (see Johnson, 1986; Berti et al., 2000).

The present study supports the conclusion that selection of relevant information in working memory is mirrored in the P3a (Berti, 2008). The P3a is pronounced in switching trials irrespective of the nature of the subsequent updating task (replacement or computation). In addition, peak latency of the P3a is virtually the same in both conditions. In contrast, the increase of switching costs in the processing condition compared with the replacement condition is not correlated with differences in the P3a obtained in these conditions. Therefore, these results suggest that the switching-related processes correlated with the P3a are a pre-requisite for subsequent selection of relevant information in working memory prior to the actual updating process and presumably also prior to selection itself. This also enhances our understanding of the processes underlying P3a generation because the P3a is typically interpreted as a correlate of—presumably automatic—allocation of attention to changes in the sensory input (for instance, in oddball-like experimental stimulation, see Escera et al., 2001; Polich, 2007). The present study supports the hypothesis that the P3a also mirrors processes of attentional allocation within working memory (Berti, 2008).

Which sub-process required to perform the updating task is correlated with the P3a? Berti (2008, 2013) and Hölig and Berti (2010) argued that the P3a component may mirror two different aspects of attentional control: (1) the disengagement or unhitching of attention from the present task (Polich, 2007) and (2) the control of attention in the service of updating of task relevant information (Barcelo et al., 2006). The *post hoc* analysis of the switching trials suggest an additional process that is required to perform this dynamic working memory updating task, namely the orientation of attention in the visual space as a pre-requisite to encode the relevant memory item and/or the particular information for the updating procedure. The results of the present study cannot distinguish between these different aspects of attentional allocation. However, the present ERP results suggest an overlap of different fronto-central components, which fits to this interpretation of different sub-processes.

Object switching is a complex phenomenon consisting of several sub-processes. In the present task, switching can be subdivided into at least three distinct processes: (1) the processing of the cue (i.e., of the location where the number or plus/minus sign is presented); (2) the allocation of attention to the relevant object or location (i.e., by activation of the representation); and (3) the preparation of the designated object for subsequent processing (i.e., making the stored representation accessible). This interpretation would match the bi-phasic ERP in the P300 time window (Berti, 2012, 2013). In both conditions, the cue is processed and the indicated object is updated irrespective of whether a switch or a repetition is required; this is mirrored in the elicitation of an early and a late positive component in both trial types. In trials where the cue indicates the selection of a new object (i.e., a switch), an additional process is required, namely the allocation of attention to either another representation in working memory or to the cue-location indicating the updating procedure. This is mirrored in the component visible in the difference waves and that overlaps with the early positive peak.

At the present stage, this tri-partite model of object switching is only a hypothesis that requires further investigation. But there are some arguments supporting this hypothesis. Firstly, the early positive peak presumably constitutes a P2 component with prolonged peak latency correlated with the processing of the location cue (for review, see Crowley and Colrain, 2004). For instance, Berti and Wühr (2012) demonstrated that a P2 was elicited when the visual stimulation contained a cue indicating the task relevant object for subsequent selection of the relevant information. This matches the present study in which a cue is required to identify the relevant object in working memory and in which the object-cue is presented together with the task-cue. Moreover, García-Larrea et al. (1992) argued that the elicitation of a P2 indicating the detection of relevant information is a pre-requisite for the elicitation of a P3a.

Secondly, Berti (2013) demonstrated that unhitching of attention may already arise at the level of the P2. In this study, either P2 or P3a was elicited by rare stimuli and it was suggested that the typical P3a findings (in the sense of novelty detection in an oddball paradigm, Friedman et al., 2001) may be a result of an overlap of these two components. In contrast, the different tasks and/or stimulus presentation characteristics in the present as well as in the studies by Berti (2012, 2013) may have resulted in a dissociation of overlapping processes and components (Debener et al., 2005).

Thirdly, the present results mirror those reported by Hölig and Berti (2010). In this study, a task switching logic was applied to an auditory oddball paradigm and it was tested whether the P3a differed between a situation in which a rare stimulus (defined by the change of the pitch of the stimulus) indicated a task switch and a situation in which no task switch is indicated. The main result was that rare stimuli elicited the P3a in both conditions but that the P3a was increased in the task switch condition. This is comparable with the present results because both studies demonstrate an increased P3a in switching-trials (task switch in Hölig and Berti, 2010, vs. object switch in the present study).

Taken together, it is plausible that the frontal positivity is a mixture of a P2-like component and the classical P3a (cf., Berti, 2012, 2013). In this line of argumentation, one may assume that the P2-like component reflects the processing of location-cue (including unhitching of attention from the current item), which indicates whether an object switch is necessary. In case the location is changed, attention has to be allocated to the indicated object in working memory; this is correlated with the P3a. This interpretation is also in line with the finding of an effect of the type of switching with regard to the distance between the different cue-locations. On the other hand, on basis of the present results it remains an open question whether the fronto-central effects in the early P300 time window can be explained by the “spatial factor” solely. However, the gradual differences between switching and repetition trials in the early time window may also suggest that both types of trials require attentional allocation because irrespective of whether a location is repeated or changed the respective memory object has to be updated. Therefore, another interpretation of the present sequence is that the P3a differences reflect that selecting a new item is more demanding than selecting a recently updated item. This is rather unlikely because this would not explain why far-switches and near-switches differ when switching trials are pooled for the two task conditions. But at present, these alternative interpretations cannot be ruled out.

To sum up, the present study supports the hypothesis that selection of relevant information in the sensory environment and in working memory share some neuro-cognitive processes (for instance, Awh and Jonides, 2001; Bledowski et al., 2010). This is also in line with the controlled attention view of working memory (Cowan, 1998; Engle et al., 1999; Oberauer, 2002; Oberauer et al., 2013) and further supports the hypothesis of strong connections between attention and working memory functions (e.g., Berti and Schröger, 2003). Interestingly, Nobre and colleagues (e.g., Griffin and Nobre, 2003; Kuo et al., 2009) demonstrated that the functional overlap of selective attention and working memory is mirrored in other ERP components, too (i.e., in the N2pc; Kuo et al., 2009). This suggests that different processes of attentional control and orientation interact in order to enable effective and flexible selection of relevant information in the environment and in working memory.

## Author Contributions

SB designed the study, analyzed the results, and wrote the manuscript.

## Conflict of Interest Statement

The author declares that the research was conducted in the absence of any commercial or financial relationships that could be construed as a potential conflict of interest.
